# Hip fracture care in Africa: Time for action

**DOI:** 10.1371/journal.pgph.0006414

**Published:** 2026-06-24

**Authors:** Sarah Drew, Nyasha Mafirakureva, Celia L. Gregson

**Affiliations:** 1 Global Health and Ageing Research Unit, Bristol Medical School, University of Bristol, Bristol, United Kingdom; 2 Sheffield Centre for Health and Related Research (SCHARR), School of Medicine & Population Health, University of Sheffield, Sheffield, United Kingdom; 3 The Health Research Unit Zimbabwe, Biomedical Research and Training Institute, Harare, Zimbabwe; PLOS: Public Library of Science, UNITED STATES OF AMERICA

## The cost of fractures

In this Decade of Healthy Ageing [1], we are seeing important demographic shifts in ageing across Africa. Age-related diseases, previously under-recognised, are manifesting substantial health demands. Fragility fractures of the hip fracture are no exception. Our Wellcome-funded Fractures-E3 research programme [2] (the 3Es being epidemiology, economics and ethnography) has demonstrated current and projected hip fracture incidence, e.g., in The Gambia hip fracture numbers are predicted to increase 4-fold in the next 30-years [3]. Costs are high, both to the patient and health system; in Zimbabwe the 71% who survive to 12-months post hip fracture have profound and sustained losses in quality of life, 97% still report pain, 98% remain disabled [4]. Outcomes may reflect the considerable average 20 days wait in public hospitals for an operation [4]. This inefficient care is expensive: the US$3,887 mean cost of admission including surgery is largely born out-of-pocket conferring catastrophic costs to patient and family [5]. Even the unacceptably high 45% managed without surgery in public hospitals (largely due to unaffordability) [4], incur mean US$1,461 out-of-pocket costs for their average 25-day admissions [5]. The total annual spending on hip fractures, obtained by applying mean costs to the estimated number of hip fractures, equates to 0.5% of healthcare spending for Zimbabwe in 2023 [6]. In South Africa, where operative management is the norm, lengths of stay are similar (mean 21 days), but costs are greater; US$6935 per patient, equating to six-times the mean per capita health expenditure for the country, and when applied to the estimated number of hip fractures, also equal to 0.5% of national healthcare spending in 2020 [7]. Assessment of fracture service availability and readiness in Zimbabwe and The Gambia has highlighted major deficits in what are complex care pathways; understaffing, lack of functioning X-ray facilities, widespread stock-outs of essential medicines, and unregulated variability in practice [8].

## Ethnographic research

To inform healthcare improvement strategies and policy reform, we undertook an extensive ethnographic study across urban and rural settings in South Africa, The Gambia and Zimbabwe. In each country we conducted up to 40 case studies with patients and caregivers, performing observations in homes and hospitals, with repeated in-depth interviews to explore care journeys, including points of disengagement and reengagement, and implications of care on wellbeing. Contemporaneous in-depth examinations of hip fracture service organisation and delivery, interviewing and observing around 40 healthcare professionals across four or five hospitals per country, added to the evidence generated. In The Gambia, where traditional bone setters remain central to fracture care, we examined their practices, including their interactions with biomedical hospital-based care, through interviews and treatment observations with 16 traditional bone setters.

Our research identifies critical gaps and opportunities to improve hip fracture care across Africa.

- **Care pathways are complex and fragmented in The Gambia,** where patients must navigate between biomedical hospital-based services and traditional bone setters. Care seeking decisions are shaped by many factors, including structural barriers within the healthcare system and financial trade-offs where resources are scarce [9].
- **In Zimbabwe systemic barriers challenge ‘responsive’ delivery of hip fracture care.** Patients and caregivers want timely and dignified care, clear communication from healthcare professionals, social support, adequate pain relief and affordable treatment. Yet understaffing, resource shortages and surgical delays often prevent needs being met [10].
- **In South Africa hip fracture service implementation and valued collaborative teamworking** are challenged by competing priorities undermining shared treatment goals. Staff shortages and limited resources mean healthcare workers frequently improvise in care delivery [11].
- **Multiple factors cause long delays to hip fracture surgery in South Africa and Zimbabwe.** Patients underestimated injury severity through lack of awareness of fragility fractures. In Zimbabwe care seeking can be delayed whilst finances are mobilized. Ambulances (in South Africa) and hospitals deprioritise hip fractures compared to other emergencies. Patients often struggle to access care and caregivers must work hard to obtain timely treatment for relatives [10,12].
- **Traditional bone setters want to collaborate with formal healthcare services.** They informally and inconsistently refer patients to hospitals for X-rays, pain relief, and other care. Some healthcare professionals believed traditional bone setters should not treat hip fractures, while others proposed better regulation of practices and training opportunities [13].

## Evidence-based recommendations to improve hip fracture care provision

**Fig 1:**
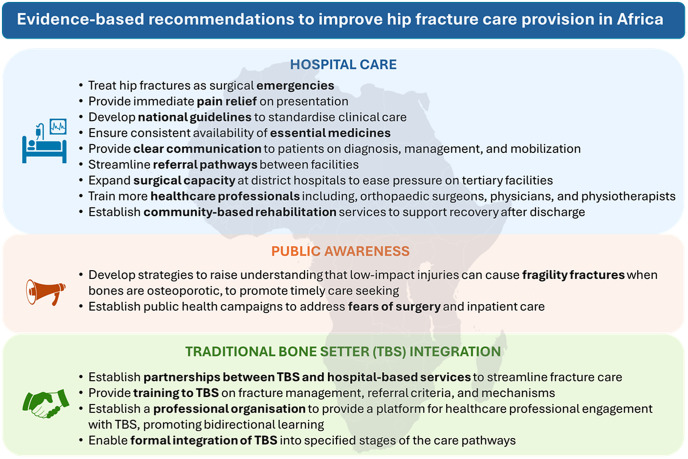
Evidence-based recommendations to improve hip fracture care provision in Africa.

Although The Gambia, South Africa and Zimbabwe represent unique contexts, all three countries share clear priorities for action (Fig 1).

### Hospital care

Hospitals should treat hip fractures as surgical emergencies, setting clear time-to-surgery targets with regular monitoring. On presentation, patients should receive immediate pain relief. National guidelines need to be developed to standardise clinical care. Essential medicines must be consistently available. Healthcare staff should communicate to patients and caregivers the diagnosis and management plans and, before discharge, advice regarding mobilisation, and home adaptation (in the absence of community rehabilitation). Referral pathways between facilities need streamlining to enable timely access to surgical care. In the longer term, surgical capacity needs to expand at district hospitals to ease pressure on tertiary facilities, alongside service development at higher-level centres. Training more healthcare professionals including orthopaedic surgeons, physicians to manage medical complexity, and physiotherapists will be essential. Community-based rehabilitation services need setting-up to support recovery after discharge and prevent long-term disability.

### Public awareness

Strategies to raise understanding that low-impact injuries can cause fractures when bones are fragile will promote timely care seeking. Following hospital service improvement, public health campaigns will need to address fears of surgery and inpatient care.

### Traditional bone setter integration

In The Gambia, where traditional bone setters out number formal health professionals, partnerships between traditional bone setters and hospital-based services would substantially streamline hip fracture care. Training on fracture management, referral criteria and mechanisms would improve traditional bone setter care. Establishing a professional organisation would provide a platform for healthcare professional engagement and *bidirectional* learning. Long-term, formal integration of traditional bone setters into specified stages of the care pathways would improve timely hospital care and pain relief. These actions would improve patient safety and strengthen trust between practitioners in ways that respect patients’ beliefs.

## Conclusion

Hip fractures are a particularly costly stressor on health services and substantially reduce quality of life. Policy and practice change is needed now to deliver timely, equitable care across Africa.
